# Photodynamic Therapy in Advanced Tracheobronchial Cancers

**DOI:** 10.1155/DTE.5.245

**Published:** 1999

**Authors:** Tom G. Sutedja

**Affiliations:** Department of Pulmonary Medicine University Hospital Vrije Universiteit P.O. Box 7057 Amsterdam 1007 MB The Netherlands

## Abstract

Photodynamic therapy (PDT) has been introduced in the early eighties for treating patients with malignancies in the tracheobronchial tract. After intravenous injection of the photosensitizers, the tumor area in the tracheobronchial tree is illuminated bronchoscopically using a laser fiber to transmit light of a specific wavelength during the procedure. Secondary tissue necrosis ensues, because of the thrombosis of the tumor vasculature leading to late tissue hypoxia. Ample data have shown that PDT is effective to obtain full depth tissue necrosis with relative sparing of the normal tissue. Local tumor control can be achieved. Competitive endoscopic techniques such as lasers and electrocautery are applicable to debulk tumor in a less selective but more immediate manner. Skin photosensitivity is a potential morbidity of PDT, especially in using the first generation photosensitizers. This limits its palliative potential. More selective and more phototoxic sensitizers in combination with the use of portable diode laser, may improve the clinical usefulness of PDT in the management of lung cancer patients. However, cost-effectiveness studies comparing PDT and other local bronchoscopic treatment modalities such as thermal lasers, electrocautery, cryotherapy, brachytherapy, whether or not in addition to external radiotherapy and chemotherapy, should be conducted to define its definite role in the palliative treatment of advanced obstructive bronchial cancers.


*Diagnostic and Therapeutic Endoscopy*, Vol. 5, pp. 245–251
Reprints available directly from the publisher
Photocopying permitted by license only

© 1999 OPA (Overseas Publishers Association) N.V.
Published by license under
the Harwood Academic Publishers imprint,
part of The Gordon and Breach Publishing Group.
Printed in Malaysia.

### Photodynamic Therapy in Advanced Tracheobronchial Cancers

TOM G. SUTEDJA*

*Department of Pulmonary Medicine, University Hospital Vrije Universiteit, P.O. Box 7057, 1007 MB Amsterdam, The Netherlands*

*(Received 18 January 1999; Revised 18 March 1999; In final form 5 April 1999)*

**Photodynamic therapy (PDT) has been introduced in the early eighties for treating patients with malignancies in the tracheobronchial tract. After intravenous injection of the photosensitizers, the tumor area in the tracheobronchial tree is illuminated bronchoscopically using a laser fiber to transmit light of a specific wavelength during the procedure. Secondary tissue necrosis ensues, because of the thrombosis of the tumor vasculature leading to late tissue hypoxia. Ample data have shown that PDT is effective to obtain full depth tissue necrosis with relative sparing of the normal tissue. Local tumor control can be achieved. Competitive endoscopic techniques such as lasers and electrocautery are applicable to debulk tumor in a less selective but more immediate manner. Skin photosensitivity is a potential morbidity of PDT, especially in using the first generation photosensitizers. This limits its palliative potential. More selective and more phototoxic sensitizers in combination with the use of portable diode laser, may improve the clinical usefulness of PDT in the management of lung cancer patients. However, cost-effectiveness studies comparing PDT and other local bronchoscopic treatment modalities such as thermal lasers, electrocautery, cryotherapy, brachytherapy, whether or not in addition to external radiotherapy and chemotherapy, should be conducted to define its definite role in the palliative treatment of advanced obstructive bronchial cancers.**

*Keywords:* Advanced lung cancer, Photodynamic therapy

### INTRODUCTION

Lung cancer is a disease of epidemic proportion and is the global cause of cancer death. Only a minority of the patients, about 13%, will be cured. Palliation is an important part of the lung cancer management due to the late stage of disease at presentation, and the high rate of recurrent and metastatic disease despite previous conventional treatments. Advanced local disease in the central airways has morbid implications [1–5]. About 30–40% of all lung cancer patients with locally advanced disease is estimated to suffer from tumor obstruction in whom external radiotherapy is considered the treatment of

* Tel.: 31-20-444 4444. Fax: 31-20-444 4328. E-mail: tg.sutedja@azvu.nl.

choice for local control. This category of patients may also be candidates for bronchoscopic treatment. Tumor obstruction causes various pulmonary symptoms: dyspnea, hemoptysis, stridor, and obstructive pneumonia. Ultimately, severe impairment of the gas exchange will lead to respiratory failure. Efforts should be undertaken to improve the quality of life despite the poor prognosis of many patients with advanced stage cancer. Bronchoscopic treatment modalities may provide symptomatic relief, enabling the bronchoscopist to perform tumor debulking [6]. Stent insertion is also possible in cases with extraluminal compression of the airway [7]. These intervention techniques have been shown to be feasible and effective, however, some limitations exist. Experience, skill and knowledge of the anatomy is important to prevent serious complications such as fistula and fatal hemorrhage. It is more difficult to treat tumor extending distal to the segmental bronchi beyond the visibility of the bronchoscopist. The depth of tumor necrosis depends not only on the treatment modality but also on technical performance. The bronchoscopist has to understand the anatomy of the tracheobronchial wall to prevent complications. The tracheobronchial tree is anatomically close to vital organs, especially pulmonary vessels accompanying its branches. This short review will deal with the role of photodynamic therapy in treating patients with locally advanced lung cancer.

### TECHNICAL CONSIDERATIONS

Photodynamic therapy (PDT) is the combined use of light and photosensitizers in the presence of oxygen to obtain tissue necrosis. Many reviews have dealt with the basic issues and mechanisms of PDT [8–11]. In treating lung cancer, patients are given sensitizers systemically by intravenous route [8,11]. The drugs used for PDT in lung cancer were mainly hematoporphyrin derivative (HpD) and its purified product di-hematoporphyrin ether (Porfimer Sodium, Photofrin II$^{R}$, QLT Vancouver Canada), developed by Dougherty [8]. Advised drug doses are 2–5 and 1–2 mg/kg respectively. After 24–48 h, sufficient sensitizers molecules are accumulated in tumor tissue. No significant drug toxicity has been reported, except for the skin photosensitivity for 6 weeks or more post-injection [12]. The drug will gradually be metabolized and photobleached. Skin phototoxicity such as skin redness and edema, has been reported to occur in 20–40% of the patients without causing any irreversible skin scarring.

More recent reports have used other more phototoxic sensitizers which interact with light of another wavelength [13]. This may optimize treatment efficacy in two ways: lower dose sensitizers may significantly reduce skin photosensitivity, while necrosis in depth is obtainable due to the deeper penetration of light with a longer wavelength. It has been argued that selective tumor damage is attributable to the selective illumination, rather than tissues' selective retention of photosensitizer molecules. Nevertheless, full regeneration of normal tissue after PDT has been shown and the fact that PDT does not damage the bronchial cartilage may prove to be advantageous in clinical practice [14]. *In vivo* studies have shown that the main effect of PDT is acute vascular thrombosis (vascular shut-off), leading to secondary hypoxia and late necrosis [15]. Complex issues remain to be investigated such as the drug pharmacokinetics, the optimal time gate between drug injection and illumination, the sensitizers degradation and depletion of oxygen during illumination procedure [8–11,16]. Tumor illumination is easy to perform. Many laser fibers are commercially available to assure homogeneous light distribution. The flexible laser fibers allow easy manipulation for treating tumor extending into the segmental bronchi. The thin laser fiber can be passed through the working channel of a fiberoptic bronchoscope [8,11]. It is better to illuminate a larger area than the suspicious lesion alone because tumor margins are difficult to delineate. Interstitial illumination are mostly used by sticking the cylindrical diffusers in the intraluminal tumor mass, enabling sideways and more homogeneous illumination along the tracheobronchial wall. Currently, cheaper and more practical diode lasers are available as light sources (see Fig. 1), which greatly improved the process of illumination compared to the previous bulky, more expensive and difficult to maintain dye laser equipments. The wavelength of light has to correlate with the absorbance characteristics of each sensitizers, to achieve optimal penetration of light photons in tissue [8,17]. This is why for HpD and DHE, light of 630 nm wavelength is used. Another interesting approach is the deliberate use of a specific wavelength, to obtain either superficial or in depth necrosis, to match the tumor thickness preventing overtreatment which may lead to cicatritial fibrosis or perforation [18]. This is particularly important in treating hollow organs to reduce the chance of perforation and extensive tissue scarring. Limiting the damage of normal parenchyma in preserving lung function is the goal of selective treatment in patients whose lung capacity is already reduced by COPD, recurrent tumor, surgical resection and radiotherapy. Light dosimetry remains one of the complex issues in PDT [8,11,16]. The ultimate tissue necrosis depends on the activation of sensitizers by light photons in the presence of oxygen. The ideal situation will be the predictability of light dose and light dose fractionation to achieve optimal necrosis in each individual patient at each particular PDT session, taking into account the individual variability of drug molecules distribution. Tumor mass in each individual case is also different in shape and size. Tracheobronchial wall is also non-homogeneous in structure. Photons distribution, absorption, scattering and reflection in tumor tissue containing sensitizers molecules are complex variables. It remains to be seen, whether a feedback monitoring during the illumination process using the real time fluorescence measurement is clinically practical to optimize tumor damage [18].

Standard protocol of illumination currently applied is in the range of 100–200 J per cm tumor

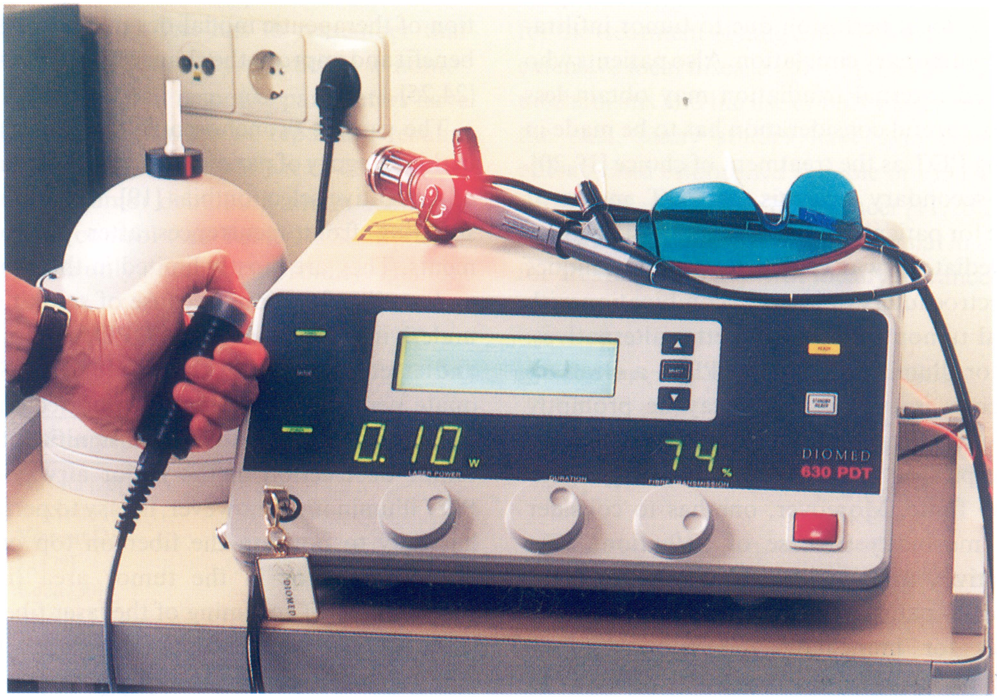

FIGURE 1 A diode laser is now available for light illumination during PDT. After patients have been given photosensitizers, the lesion is illuminated using special laser fiber which transmits the light homogenously around or in the tumor area.

length axis [8,11]. Bronchoscopic PDT for tumor obstruction in the large airways does not allow long duration of illumination due to the poor condition of many patients. Treatment modalities such as PDT, especially when performed under local anesthesia have to be limited to 15–20 min to minimize the risk of respiratory failure. Secondary necrosis and formation of fibrin plugs require prompt action for a "clean-up" bronchoscopy to remove debris. If necessary, re-illumination may be performed. Afterwards, further considerations should be made whether patients should receive additional palliative treatment [6].

### PATIENTS SELECTION

Respiratory failure may already be imminent if there is a significant tumor obstruction of the central airways. Bulky peribronchial disease cannot be treated solely by any debulking technique. Reopening the airway lumen does not always result in the improvement of gas exchange if there is a significant reduction of local perfusion due to tumor infiltration in the pulmonary circulation. Also patients who had received external irradiation may obtain less benefit. So, careful consideration has to be made in considering PDT as the treatment of choice [19,20]. The late secondary necrosis of PDT makes it unsuitable for patients with severe tracheal obstruction. Immediate airway clearance using techniques such as electrocautery or lasers in combination with mechanical tumor removal are better alternatives. After tumor illumination during PDT, a clean-up bronchoscopy is essential and may be promptly required for removal of fibrin plugs and necrosis to prevent respiratory failure [8,11]. Light penetration is also finite. Moreover, one has to consider treatment morbidity in case of palliation. Skin photosensitivity may well be less burdensome for the individual patient, however, there are several alternatives for immediate and non-immediate tumor debulking in case of tumor obstruction in the tracheobronchial tree [6].

Thermal lasers such as Nd-YAG laser and electrocautery can obtain a more immediate effect in a less selective way. Cryotherapy is a cheaper technique also causing secondary effect and is equally safe for bronchial cartilage [21]. High dose rate brachytherapy is technically a sophisticated method of intraluminal irradiation with short treatment time, but damage to normal tissue may lead to radiation bronchitis, scarring and fistula [22]. It is therefore important to compare the cost effectiveness of PDT and other bronchoscopic techniques in patients with advanced lung cancer. Many patients with advanced lung cancer will succumb from metastatic disease. PDT and other treatment modalities may provide long duration of local control, while sparing normal lung parenchyma as much as feasible. Local control and distant control are not independent factors in lung cancer care [23]. There is virtually no improvement of quality of life when neither local nor distant control can be achieved. Both local and distant tumor controls are equally important. Long duration of symptomatic relief does improve quality of life of patients suffering from end-stage cancer. Looking for new avenues for the optimal application of therapeutic modalities to provide long term benefit and improve the chance for cure is warranted [24,25].

The various bronchoscopic treatment modalites in this category of patients share the similar problem of a negative selection bias [19]. Many patients are suffering from recurrences after previous treatments. They are mostly limited in their pulmonary and cardiac functions because of smoking history, underlying diseases such as COPD, previous surgery and external radiation therapy [3,6,19]. So, inadequate performance of intervention techniques can worsen patients' condition if significant residual tumor obstruction in the major airways persists. PDT illumination, however, is easy to perform. One has only to position the fiber on top of (surface illumination), or in the tumor area (interstitial illumination). The aiming of the laser fiber is much less risky and requires less manipulation than Nd-YAG laser or electrocautery. Secondary necrosis requires clean-up bronchoscopy for removal of tissue debris and fibrin plugs. The bronchoscopist has to avoid respiratory failure and may use the opportunity to re-illuminate the tumor. Patients have to be protected from UV light (sunlight) after sensitizers have been injected. Photosensitizers are retained in the skin for a period of 6 weeks or more, and only gradually being metabolized and photobleached [8,11,12].

PDT may provide an option to reduce intraluminal tumor extent enabling a less extensive surgical resection afterwards [24]. PDT and other bronchoscopic treatment modalities should be part of the treatment strategy within the framework of lung cancer care in combination with surgery, chemotherapy and radiotherapy. This multidisciplinary approach focuses on both palliation and treatment with curative intent [6,8,11,20,24,25].

### CLINICAL RESULTS

Several PDT phase II and phase III studies have been published [26–33]. In general, they all showed that recanalization of the tracheobronchial tree can be achieved, irrespective of the previous treatments given to the patients. One has to remember that locally advanced cancer is by no means a homogeneous disease and that response parameters have been used in different ways in reporting results. Nevertheless, PDT has been shown to be effective for intraluminal tumor debulking and in achieving long term local control (see Table I). Skin photosenstitivity is the only potential morbidity. Hemorrhages were reported after PDT and might be related to the negative selection bias of patients' population. Bulky tumor might have infiltrated the big vessels around the tracheobronchial tree prior to treatment. Tumor recurrences after conventional treatments such as surgery and external radiotherapy are often the tip of an iceberg. New data slowly emerge in using more phototoxic photosensitizers such as m-THPC in treating early lung cancers [18]. The availability of portable diode laser in compact format (see photograph) makes PDT more practical to perform, but more phase III studies are needed to compare the cost-effectiveness of various palliative bronchoscopic treatments. Whether the use of the new generation sensitizers in combination with the diode laser may become more popular for palliation and become competitive to other techniques, remains to be seen.

### FUTURE PROSPECTS

Ample data have shown the clinical efficacy of PDT for the treatment of lung cancer, also in patients with extensive local disease [26–33]. Despite its complex mechanisms and the use of bulky laser equipment in the past, many studies have also confirmed its efficacy and established the role of PDT in treating superficial early stage lung cancer [11,20,24,25,34].

The issue is thus more controversial in using PDT for treating patients with locally advanced cancers

TABLE I Response of palliation with PDT in patients' subgroups with advanced stage and obstructive lung tumors

| References [no.] | *n* | Response | Survival (months) |
|---|---|---|---|
| Hayata [26] | 14 | 63% | Up to <16 |
| Balchum [27] | 81 | 99% | Median <7 |
| Vincent [28] | 17 | 76% | Median 1.5 |
| Li [29] | 21 | 83% | Not available |
| Edell [30] | 38 | 68% | 3–53 |
| Sutedja [31] | 26 | 73% | Median 4 |
| *Randomized studies* | | | |
| Lam [32] RT alone vs PDT + RT | 22 | PDT + RT gave longer remission | 4–10 |
| Moghissi [33] PDT vs Nd-YAG | 26 | PDT: better response and lung function improvement | Not available |

Abbreviations: *n* = number of patients in the study; RT = radiotherapy.

[6,7,19,20,35]. The secondary mechanism of tumor necrosis, the availability of other treatment modalities that can be applied for immediate palliation and the cost-effectiveness issue are factors to be considered before choosing PDT for palliation. Less skin phototoxic second generation sensitizers and the availabilty of portable diode lasers can certainly improve PDT application. Many centers performing PDT also have Nd-YAG laser or electrocautery facilities. As immediate results can be obtained and concurrent placement of a stent is made possible, the preference for these alternative techniques are obvious. PDT achieves secondary or non-immediate necrosis and is therefore comparable to techniques such as high dose rate brachytherapy and cryotherapy [6]. The choice of treatment is also based on the patients' population referred to each particular institution.

In summary, it remains to be seen whether PDT using new photosensitizers in combination with diode lasers may provide a cost-effective alternative for bronchoscopic treatment in patients with locally advanced lung cancer.

### References

[1] Benfield, J.R. The lung cancer dilemma. *Chest* 1991; **100**: 510–511.

[2] Pearson, F.G. Current status of surgical resection for lung cancer. *Chest* 1994; **106**: 337S–339S.

[3] Pairolero, P.C., Williams, D.E., Bergstrahl, E.J. *et al.* Postsurgical stage I bronchogenic carcinoma: morbid implications of recurrent disease. *Ann. Thor. Surg.* 1984; **38**: 331–338.

[4] Majid, D.A., Lee, S., Kushalani, S. *et al.* The response of atelectasis from lung cancer to radiation therapy. *Int. J. Rad. Oncol. Biol. Phys.* 1986; **12**: 231–232.

[5] Chetty, K.G., Moran, E.M., Sasoon, C.S. *et al.* Effect of radiation therapy on bronchial obstruction due to bronchogenic carcinoma. *Chest* 1989; **95**: 582–584.

[6] Sutedja, G. and Postmus, P.E. Bronchoscopic treatment of lung tumors. Review article. *Lung Cancer* 1994; **11**: 1–17.

[7] Dumon, J.F. A dedicated tracheobronchial stent. *Chest* 1990; **97**: 328–332.

[8] Dougherty, T.J. Photodynamic therapy – new approaches. *Sem. Surg. Oncol.* 1989; **5**: 6–16.

[9] Ash, D. and Brown, S.B. Photodynamic therapy – achievements and prospects. *Br. J. Cancer* 1989; **60**: 151–152.

[10] Gomer, C.J., Rucker, N., Ferrario, A. *et al.* Review. Properties and applications of photodynamic therapy. *Rad. Res.* 1989; **120**: 1–18.

[11] Sutedja, G. and Postmus, P.E. Photodynamic therapy in lung cancer. A review. *J. Photochem. Photobiol.* 1996; **36**: 199–204.

[12] Dougherty, T.J., Cooper, M.T. and Mang, T.S. Cutaneous phototoxic occurrences in patients receiving Photofrin. *Lasers in Surgery and Medicine* 1990; **10**: 485–488.

[13] Grosjean, P., Savary, J.F., Wagnieres, G. *et al.* Tetra (*m*-hydroxyphenyl) chlorin clinical photodynamic therapy of early bronchial and oesophageal cancers. *Laser Med. Sci.* 1996; **4**: 227–235.

[14] Smith, S.G.T., Bedwell, J., MacRobert, A.J. *et al.* Experimental studies to assess the potential of photodynamic therapy for the treatment of bronchial carcinomas. *Thorax* 1993; **48**: 474–480.

[15] Star, W.M., Marijnissen, H.P., vdBerg Blok, A.E. *et al.* Destruction of rat mammary tumor and normal tissue microcirculation by hematoporphyrin derivative photoradiation observed *in vivo* in sandwich observation chamber. *Cancer Res.* 1986; **46**: 2532–2540.

[16] Van Gemert, J.C., Berenbaum, M.C., Gijsbers, G.H. *et al.* Wavelength and light-dose dependence in tumor phototherapy with hematoporphyrin derivative. *Br. J. Cancer* 1985; **52**: 43–39.

[17] Sliney, D.H. Laser-tissue interactions. *Clinics in Chest Medicine* 1985; **2**: 203–208.

[18] Braichotte, D., Savary, J.F., Glanzman, T. *et al.* Optimizing light dosimetry in photodynamic therapy of the bronchi by fluorescence spectroscopy. *Lasers Med. Sci.* 1996; **11**: 247–254.

[19] Dumon, J.F., Shapsay, S., Bourceraou, J. *et al.* Principles for safety in application of neodymium-YAG laser in bronchology. *Chest* 1984; **86**: 163–168.

[20] Edell, E.S., Cortese, D.A. and McDougall, J.C. Ancillary therapies in the management of lung cancer: photodynamic therapy, laser therapy and endobronchial prosthetic devices. *Mayo Clin. Proc.* 1993; **68**: 685–690.

[21] Homasson, J.P. Bronchoscopic cryotherapy. *J. Bronchology* 1995; **2**: 145–153.

[22] Villanueva, A.G., Lo, T.C. and Beamis, J.F. Endobronchial brachytherapy. *Clinics in Chest Medicine* 1995; **16**: 445–454.

[23] LeChevalier, M., Arriagada, R., Cuoix, E. *et al.* Radiotherapy alone versus combined chemotherapy and radiotherapy in nonresectable non-small cell lung cancer – first analysis of a randomized trial in 353 patients. *J. Natl. Cancer Inst.* 1991; **83**: 417–423.

[24] Kato, H., Konaka, C., Ono, J. *et al.* Preoperative laser photodynamic therapy in combination with operation in lung cancer. *J. Thorac. Cardiovasc. Surg.* 1985; **90**: 420–429.

[25] Okunaka, T., Kato, H., Konaka, C. *et al.* Photodynamic therapy for multiple primary bronchogenic carcinoma. *Cancer* 1991; **68**: 253–258.

[26] Hayata, Y., Kato, H., Konaka, C. *et al.* Hematoporphyrin derivative and laser photoradiation in the treatment of lung cancer. *Chest* 1982; **81**: 269–277.

[27] Balchum, O.J., Doiron, D.R. and Huth, G.C. Photoradiation therapy of endobronchial lung cancer. Large obstructing tumors, non-obstructing tumors and early stage bronchial cancer lesions. *Clin. Chest Med.* 1985; **6**: 255–275.

[28] Vincent, R.G., Dougherty, T.J., Rao, U. *et al.* Photoradiation therapy in advanced carcinoma of the trachea and bronchus. *Chest* 1984; **85**: 29–33.

[29] Li, J.H., Chen, Y.P., Zhao, S.D. *et al.* Application of hematoporphyrin derivative and laser-induced photodynamic therapy in the treatment of lung cancer. *Lasers Surg. Med.* 1984; **4**: 31–37.

[30] Edell, E.S. and Cortese, D.A. Bronchoscopic phototherapy with hematoporphyrin derivative for treatment of localized bronchogenic carcinoma: a five-year experience. *Mayo Clin. Proc.* 1987; **62**: 8–14.

[31] Sutedja, G., Baas, P., Stewart, F. *et al.* A pilot study of photodynamic therapy in patients with inoperable non-small cell lung cancer. *Eur. J. Cancer* 1992; **28A**: 1370–1373.

[32] Lam, S., Kostashuk, E.C. and Coy, P. A randomized comparative study of the safety and efficacy of photodynamic therapy using Photofrin II combined with palliative radiotherapy versus palliative radiotherapy alone in patients with inoperable obstructive bronchogenic carcinoma. *Photochem. Photobiol.* 1987; **46**: 893–897.

[33] Moghissi, K., Dixon, K. and Parsons, R.J. A controlled trial of Nd-YAG laser vs photodynamic therapy for advanced malignant bronchial obstruction. *Lasers Med. Sci.* 1993; **8**: 269–273.

[34] Hayata, Y., Kato, H., Konaka, C. *et al.* Photodynamic therapy (PDT) in early stage lung cancer. *Lung Cancer* 1993; **9**: 287–294.

[35] Cavaliere, S., Foccoli, P. and Farina, P.L. Nd:YAG laser bronchoscopy. A five-year experience with 1,396 applications in 1000 patients. *Chest* 1988; **94**: 15–21.

